# Hypoxia, Metabolic Reprogramming, and Drug Resistance in Liver Cancer

**DOI:** 10.3390/cells10071715

**Published:** 2021-07-06

**Authors:** Macus Hao-Ran Bao, Carmen Chak-Lui Wong

**Affiliations:** 1 Department of Pathology, The University of Hong Kong, Hong Kong, China; macusbao@gmail.com; 2 State Key Laboratory of Liver Research, The University of Hong Kong, Hong Kong, China

**Keywords:** hypoxia, metabolism, metabolic reprogramming, drug resistance, TKIs, ICIs, liver cancer

## Abstract

Hypoxia, low oxygen (O_2_) level, is a hallmark of solid cancers, especially hepatocellular carcinoma (HCC), one of the most common and fatal cancers worldwide. Hypoxia contributes to drug resistance in cancer through various molecular mechanisms. In this review, we particularly focus on the roles of hypoxia-inducible factor (HIF)-mediated metabolic reprogramming in drug resistance in HCC. Combination therapies targeting hypoxia-induced metabolic enzymes to overcome drug resistance will also be summarized. Acquisition of drug resistance is the major cause of unsatisfactory clinical outcomes of existing HCC treatments. Extra efforts to identify novel mechanisms to combat refractory hypoxic HCC are warranted for the development of more effective treatment regimens for HCC patients.

## 1. Introduction

Hepatocellular carcinoma (HCC), the most common liver malignancy (70–90%) arising from aberrant hepatocyte transformation, is the fifth-most prevalent cancer worldwide with more than 840,000 new cases and over 780,000 deaths in 2018 [1]. Due to the asymptomatic nature of HCC at early stage, most patients are diagnosed at advanced stage with limited therapeutic options. Intratumoral hypoxia, low oxygen (O_2_) level, is a crucial feature in all solid tumors, especially HCC. Hypoxia elicits a cascade of metabolic changes for hypoxic adaptation in HCC cells mostly via hypoxia-inducible factors (HIFs), the master transcription factors for hypoxic response. While hypoxia leads to drug resistance in HCC [2,3], the underlying molecular mechanisms remain largely elusive. Sorafenib was the first FDA-approved tyrosine kinase inhibitor (TKI) for first-line HCC treatment but patients quickly acquired drug resistance after three months [4,5]. Other TKIs, including Lenvatinib and Cabozantinib (first-line) as well as Regorafenib and Ramucirumab (second-line), were used as HCC palliative treatments but the survival benefits were modest [6,7,8,9,10]. Recently, immune checkpoint inhibitors (ICIs), Nivolumab and pembrolizumab were approved by FDA as a second-line treatment strategy. Tumor microenvironment (TME) is immunosuppressive with anti-tumoral immune cells, including CD8+ cytotoxic T cells and natural killer (NK) cells being exhausted and increased number of protumoral immune cells, including M2-like macrophages, regulatory T cells (Treg) and myeloid-derived suppressor cells (MDSC) [11,12]. PD-1 is an immune checkpoint suppressing immune cell activities to prevent overactivation of immune system upon binding with its ligand, PD-L1. Cancer cells hijack this pathway to evade immune surveillance by expressing PD-L1 [13]. Only 20% HCC patients were responsive to Nivolumab, an anti-PD-1 monoclonal antibody that inhibits PD-1/PD-L1 ligation [9]. Drug resistance in HCC profoundly impedes long-term clinical benefits of existing treatments. Therefore, there is an urgent clinical need to overcome drug resistance in refractory HCC. We will discuss the role of hypoxia-mediated metabolic reprogramming, which contributes to drug resistance of TKIs and ICIs, and the potential of combining drugs targeting the associated genes with TKIs and ICIs to overcome drug resistance in hypoxic HCC.

## 2. Hypoxic Tumor Microenvironment in HCC

**Figure 1:**
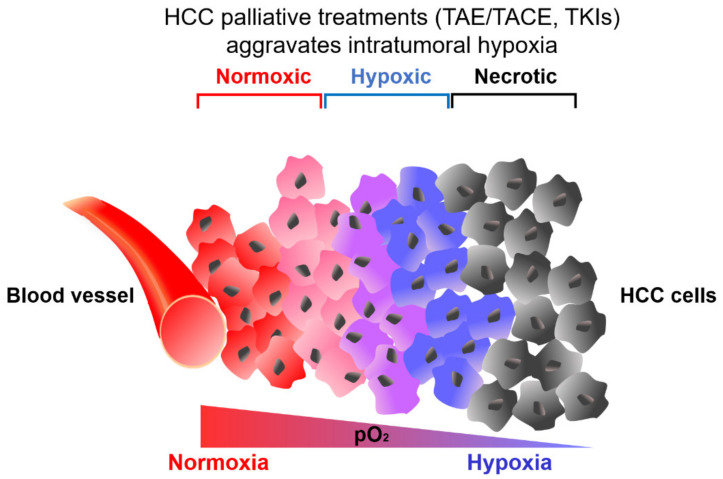
Hypoxic tumor microenvironment (TME) in hepatocellular carcinoma (HCC). A gradually decreasing gradient of partial pressure of O_2_ (pO_2_) in HCC from the blood vessel. Tumor regions that are close to the blood vessel are more oxygenated whereas regions away from the blood vessel are hypoxic. HCC treatments including arterial embolization transcatheter (TAE), arterial chemoembolization (TACE) and tyrosine kinase inhibitors (TKIs) inadvertently induced hypoxia in HCC.

HCC is one of the most hypoxic malignancies [14,15]. The median partial pressure of O_2_ (pO_2_) in normal human liver tissue is 30 mmHg while intratumoral region of human liver tumors is merely 6 mmHg [14,15]. 74.1% HCC tumor regions in rat had low pO_2_ ranging from 0 to 10 mmHg [16]. Another study in rat HCC model showed that while pO_2_ in nontumorous liver tissues reached 45 mmHg, O_2_ tension in HCC tissues was only 0.2 to 0.8 mmHg [17]. Furthermore, IHC studies revealed significantly higher expression of hypoxic markers, including HIF-1α [18], glucose transporter type 1 (GLUT1) [19], lactate dehydrogenase A (LDHA) [20] and carbonic anhydrase 9 (CA9) [21] in human HCC tissues when compared to nontumorous liver tissues. These data consolidate that HCC is highly hypoxic. Intratumoral hypoxia is caused by an imbalance of O_2_ availability due to insufficient blood supply from poor tumor vasculature, and increased O_2_ consumption by metabolic active HCC cells. HCC cells experience a continuum of O_2_ concentration gradient as tumor regions distant from the blood vessel become increasingly hypoxic (Figure 1). While tumor area with close proximity with the blood vessel is oxygenated, O_2_ level gradually decreases away from blood vessels, forming hypoxic regions [22]. Necrotic regions at the tumor core are severely hypoxic. Hypoxia in HCC is dynamic. Spatial pattern and intensity of hypoxia vary temporally according to heterogeneity of O_2_ level in the TME. Moreover, HCC treatment, transcatheter arterial embolization transcatheter (TAE) and arterial chemoembolization (TACE), which initially attempted to restrict blood supply to mitigate tumor growth, inadvertently aggravates hypoxia [23]. Tyrosine kinase inhibitors (TKIs) also aggravate intratumoral hypoxia by inhibiting multiple kinase targets, including vascular endothelial growth factor (VEGF), platelet-derived growth factor (PDGF) and fibroblast growth factor (FGF), that otherwise promote angiogenesis [21]. Hypoxia induces a series of alterations of metabolic pathways via HIFs that promotes drug resistance, leading to unsatisfactory clinical outcomes in HCC patients.

## 3. HIF-Induced Metabolic Reprogramming under Hypoxia and Drug Resistance in HCC

**Figure 2:**
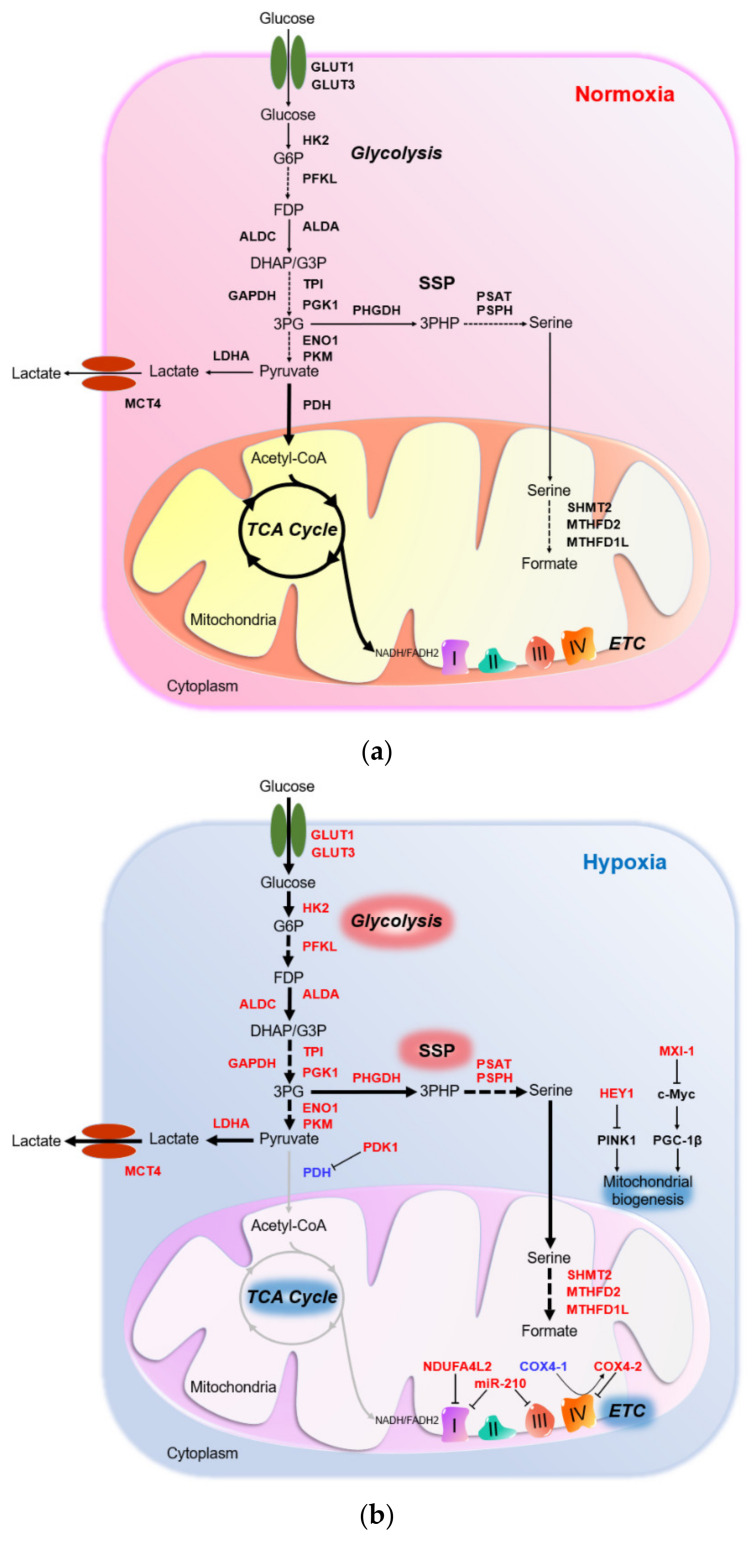
Hypoxia-inducible factors (HIFs) divert metabolites from tricarboxylic acid cycle (TCA) cycle to glycolysis under hypoxia. (**a**) Under normoxia, glucose is converted to pyruvate during glycolysis. Pyruvate is then converted to acetyl coenzyme A (acetyl-CoA), which fuels the TCA cycle for maximum adenosine triphosphate (ATP) production with ample oxygen (O_2_) supply. (**b**) Under hypoxia, metabolism is switched from oxidative to glycolytic metabolism by HIF-dependent upregulation of pyruvate dehydrogenase kinase 1 (PDK1) and (lactate dehydrogenase A) LDHA. Lactate export is promoted to prevent excessive intracellular lactate accumulation, which may lead to cytoplasmic acidification. Serine synthesis pathway (SSP) and its downstream folate cycle are activated. Folate cycle produces a major antioxidant, nicotinamide adenine dinucleotide phosphate (NADPH), to counteract oxidative stress under hypoxia. Mitochondrial activity and biogenesis are suppressed to reduce mitochondrial reactive oxygen species (ROS) production. Genes or pathways highlighted in red: upregulated by HIFs. Genes or pathways highlighted in blue: downregulated by HIFs.

**Figure 3:**
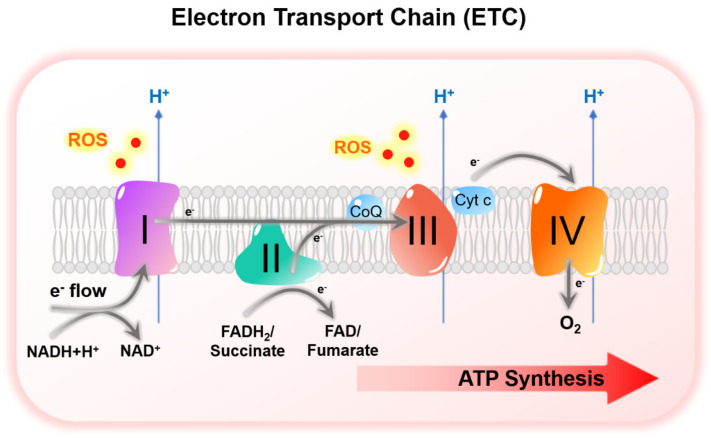
Electron transport chain (ETC). ETC is located at mitochondrial inner membrane (MIM). Electron donors produced from glycolysis and TCA cycle, nicotinamide adenine dinucleotide (NADH) and flavin adenine dinucleotide (FADH2), and succinate (glucose intermediate) donate electrons to the ETC. Electrons pass through ETC complex I to IV and finally to O_2_, the final electron accepter. Electron flow drives H^+^ export to the intermembrane space, creating a transmembrane electrical potential to drive ATP synthesis. Premature electron leakage leads to ROS accumulation, especially at complex I and complex III.

Under normoxia, glucose is broken down to pyruvate during glycolysis. Subsequently, pyruvate is converted to acetyl coenzyme A (acetyl-CoA), which enters tricarboxylic acid cycle (TCA) cycle to produce electron donors, nicotinamide adenine dinucleotide (NADH) and flavin adenine dinucleotide (FADH2) (Figure 2A). Electrons pass along electron transport chain (ETC) at mitochondria, and eventually to O_2_, the final electron acceptor to produce adenosine triphosphate (ATP) (Figure 3). This process is oxidative phosphorylation (OXPHOS). Normoxic cells exhibit high OXPHOS activity to maximize energy production for growth and proliferation. Under hypoxia with limited O_2_, electron transfer at ETC is not efficient due to the lack of final electron acceptor, leading to electron leakage and high reactive oxygen species (ROS) production from mitochondria. Excessive ROS accumulation results in irreversible oxidative damage of cellular components, eventually leading to cell death. Under hypoxia, HIFs upregulated pyruvate dehydrogenase kinase 1 (PDK1) to inhibit PHD activity, thus inhibiting conversion of pyruvate to acetyl-CoA [24,25]. At the same time, HIFs upregulated LDHA to promote conversion of pyruvate to lactate [26]. HIF-mediated *PDK1* and *LDHA* transactivation leads to the switch from oxidative to glycolytic metabolism [27] (Figure 2B), thus preventing deleterious buildup of mitochondrial ROS under hypoxia. Furthermore, glycolytic metabolite, 3-phosphoglyceric acid (3PG), enters serine synthesis pathway (SSP) generating serine, which enters the folate cycle to provide a source of nicotinamide adenine dinucleotide phosphate (NADPH) to counteract ROS. Interestingly, enzymes in the SSP and folate cycle are consistently induced by hypoxia/HIFs [28]. Since induction of oxidative stress is an important mechanism for antitumoral effect of TKIs [29], metabolic rewiring under hypoxia contributes to drug resistance by lowering ROS in TKI-treated HCC. Moreover, levels of metabolites, including glucose, lactate and adenosine were altered in TME that collectively shape an immunosuppressive environment to greatly hinder the efficacy of ICIs in HCC.

### 3.1. HIF-Mediated Induction of Glucose Metabolism under Hypoxia

Glucose uptake and glycolysis are activated in hypoxic HCC cells. HIF-1 induces the expression of solute carrier family 2 member 1 (*SLC2A1*) and solute carrier family 2 member 3 (*SLC2A3*), which encode GLUT1 and GLUT3 respectively, to promote glucose uptake to meet the insatiable demand of glucose for growth of hypoxic cancer cells [30] (Figure 3) (Table 1). PET-CT imaging found that uptake of fluorodeoxyglucose (FDG), a glucose analog, increased with decreasing O_2_ availability in cancer cells [31]. Human HCC with high FDG uptake had significantly higher expression of GLUT1 and GLUT3, and these patients had shorter overall survival [32]. Most glycolytic genes, including hexokinase (*HK*), phosphofructokinase, liver type (*PFKL*), aldolase (*ALD*), triosephosphate isomerase 1 (*TPI*), glyceraldehyde-3-phosphate dehydrogenase (*GAPDH*), phosphoglycerate kinase (*PGK*), enolase 1 (*ENO1*) and pyruvate kinase M1/2 (*PKM*), were induced by hypoxia and/or HIF-1 (Table 1) [30]. HK is the first rate-limiting enzyme of glycolysis (Figure 3). While normal hepatocytes express HK4, HK2 isoform is predominantly overexpressed in HCC [33]. The protein level of HK2 was significantly increased in multiple HCC cell lines cultured in hypoxia [34]. HK2 upregulation was mediated by HIF-1 [34]. Alternatively, HIF-1 mediated suppression of *miR-199a*, which otherwise targets HK2 for its downregulation, to induce HK2 expression in hypoxic HCC cells [35]. Upregulation of HK2 promoted glycolysis and increased lactate secretion in hypoxic HCC cells. GLUT1/3 and HK2 are prognostic markers in HCC patients. Clinically, overexpression of GLUT1/3 and HK2 was associated with poor clinical outcomes, including more advanced tumor stage, greater tumor burden, higher rate of recurrence and poor survival in HCC patients [19,32,36].

#### 3.1.1. HIF-Mediated Induction of Glucose Metabolism under Hypoxia and TKI Resistance

Drug resistant cancer cells relied heavily on glucose metabolism for survival under hypoxia [60]. HIF-mediated glycolysis contributes to Sorafenib resistance in multiple HCC cell lines [61]. GLUT1, GLUT3 and HK2 were overexpressed in Sorafenib resistance HCC cells [40,61,62,63]. Sorafenib-resistant HCC cells (Huh-7R) had markedly higher glucose uptake and lactate production rates, indicative of augmented glycolysis [61,64]. Previous studies demonstrated that activation of glucose metabolism under hypoxia is a targetable vulnerability to re-sensitize drug resistant HCC cells. 2-Deoxyglucose (2-DG), a glycolysis inhibitor, significantly potentiated toxicity of Sorafenib by reducing cell viability, inhibiting colony formation and promoting G0/G1 cell cycle arrest of Sorafenib resistant HCC cells [64]. Importantly, 2-DG synergized with Sorafenib to markedly induce apoptosis of Sorafenib resistant HCC cells (Table 2) [63]. Inhibition of another important glycolytic enzyme, HK2, by 3-bromopyruvate (3-BP) also greatly improved the efficacy of Sorafenib in hypoxic HCC cells (Table 2) [40]. Combination treatment of 3-BP and Sorafenib significantly attenuated HCC growth, demonstrating the importance of glycolysis activation for drug resistance in hypoxic HCC [40].

#### 3.1.2. HIF-Mediated Induction of Glucose Metabolism under Hypoxia and ICI Resistance

Intense nutrient competition, including glucose, between hypoxic cancer cells and anti-tumor immune cells, which both have high demand for glucose, is a common phenomenon in HCC that contributes to ICI resistance. Cancer cells often outcompete immune cells for glucose [66]. Cancer cells deplete glucose in the tumor and glucose level at TME is 3–10 times lower than that of normal tissues [67]. GLUT1 expression was highly upregulated in activated T cells and NK cells [68,69]. These anti-tumoral immune cells have high glucose demand to fuel glycolysis for their cytotoxicity [68,70]. Therefore, glucose-deprived TME is immune suppressive. CD8^+^ T cells isolated from highly glycolytic tumors with low glucose availability at TME had significantly lower rate of glycolysis, produced significantly less interferon gamma (IFNγ) and these exhaustive phenotypes were associated with faster tumor progression [66]. Recently, exhausted T cells were further categorized into progenitor exhausted T cells and terminally exhausted T cells, as defined as PD1+TIM3-TCF1+ and PD1+TIM3+TCF1- T cells respectively [71]. Interestingly, it has been recently shown that hypoxia drove the differentiation of mouse progenitor exhausted T cells to terminally exhausted T cells in vitro [72]. While these two subsets of exhausted T cells await further characterization in HCC, another emerging question that merits further exploration is whether hypoxia drives exhausted T cell differentiation through HIF-mediated transcription of genes or hypoxia-induced metabolites in T cells. Inhibition of glucose uptake in NK cells greatly impeded their effector function with lower IFNγ and granzyme B production as well as disrupted adhesion with target cells for clearance [73]. Since glucose metabolism is important for proper function of CD8^+^ T cells and NK cells, it is reasonable to speculate that glucose-deprived TME or glycolysis inhibitor is likely to hinder the efficacy of ICIs in HCC. Indeed, 2-DG inhibited T cell and NK cell growth and activity [68,73]. More pre-clinical studies are urgently needed to determine the effectiveness and therapeutic window of glycolysis inhibitors in combination with ICIs in hypoxic HCC.

### 3.2. HIF-Mediated Induction of Lactate Metabolism under Hypoxia

LDHA, which is responsible for the conversion of pyruvate to lactate, was overexpressed in hypoxic HCC cells in a HIF-1-dependent manner [26] (Figure 3) (Table 1). Excessive intracellular lactate accumulation leads to cytoplasmic acidification, which is deleterious to cell viability. To maintain normal intracellular pH, monocarboxylate transporter 4 (MCT4), a lactate exporter, is induced by both HIF-1 and HIF-2 for extrusion of lactate [41,42] (Figure 3) (Table 1). While lactate level in normal tissues ranges from 1.5–3 mM, it surges to 10–30 mM in tumor tissues [74]. Conventionally, lactate was regarded as a metabolic waste. Interestingly, the differential expression of MCT1, responsible for lactate uptake, and MCT4 within a tumor enables metabolic symbiosis between hypoxic cancer cells at tumor core and normoxic cancer cells at tumor periphery [75]. While hypoxic cancer cells preferentially consume glucose and actively secret lactate to the environment by overexpressing MCT4, normoxic cancer cells predominantly overexpress MCT1 to consume the imported lactate, later converted to pyruvate by LDH, to fuel OXPHOS for growth. Inhibition of MCT1 forced normoxic cancer cells to switch from lactate-fueled to glycolysis-dependent OXPHOS, thus depleting glucose from TME and leading to extensive cell death of hypoxic cancer cells, as they relied heavily on glucose [75]. Furthermore, reduced expression of LDHA and MCT4 effectively suppressed growth of hypoxic tumor in vivo [76,77]. Overexpression of LDHA and MCT4 were frequently found in human HCC with poor clinical outcomes, and LDHA and MCT4 may serve as independent diagnostic biomarkers in HCC [20,43].

#### HIF-Mediated Induction of Lactate Metabolism and ICI Resistance

**Figure 4:**
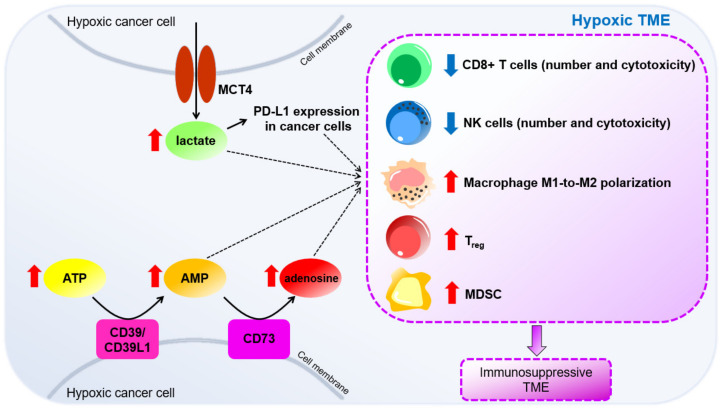
HIF-induced metabolic reprogramming under hypoxia creates an immunosuppressive TME. HIF-mediated induction of lactate metabolism and adenosinergic metabolism leads to the accumulation of oncometabolites, including lactate, adenosine monophosphate (AMP) and adenosine at TME that inhibits anti-tumoral immune cells and promotes expansion of protumoral immune cells, resulting in an immunosuppressive TME that aids immune evasion of tumor cells.

Exogenous lactate increased PD-L1 expression in lung cancer cells that was mediated via lactate receptor, G protein-coupled receptor 81 (GPR81) [78] (Figure 4). Knockdown of *GPR81* significantly inhibited lactate-induced PD-L1 expression [78]. Lactate-activated GPR81 contributed to nuclear translocation of a transcriptional coactivator, WW domain containing transcription regulator (TAZ), which then forms a complex with transcriptional enhanced associate domain (TEAD), a transcription factor that promotes PD-L1 expression [78].

In CD8^+^ T cells, lactate induced apoptosis, inhibited proliferation, decreased IFNγ production, intracellular perforin and granzyme-B levels [79] (Figure 4). High lactate level at TME inhibited lactate efflux and promoted lactate influx, leading to the accumulation of cytoplasmic lactate, causing intracellular acidification and reduction of CD8^+^ T cell viability [79]. Removal of lactate from culture medium significantly restored cytokine production and cytotoxicity of CD8^+^ T cells [79], suggesting that lactate hindered proper function of CD8^+^ T cells. Similarly, lactate induced apoptosis, reduced perforin, granzyme B production and suppressed expression of activating receptor, NKp46, in NK cells [80] (Figure 4). Lactate-treated NK cells also exhibited intracellular acidification [80]. Additionally, in mice tumor associated macrophage, lactate promoted a shift from anti-tumoral M1 phenotype to protumoral M2 phenotype in a HIF-1-dependent manner [81] (Figure 4). Moreover, lactate induced other M2 markers, including CD206 and CCL17, by activating G protein-coupled receptor 132 (Gpr132) responsible for extracellular lactate sensing [82]. Importantly, only lactate, but not low pH or M2 macrophage activating cytokine, IL-4, activated Gpr132 signaling to promote M1-to-M2 switch in macrophage [82]. M2 polarization was abrogated upon removal of lactate from culture medium [82]. Progression of cancer with high lactate content was significantly halted in *Gpr132* knockout mice with reduced tumor-associated M2 macrophages, indicating the importance of lactate/Gpr132 axis in inducing M2 macrophages [82]. Moreover, lactate increased the proportion of intratumoral immunosuppressive cells, Treg and MDSCs [83,84] (Figure 4). Interestingly, intratumoral Treg overexpressed MCT1 to take up lactate from TME to sustain OXPHOS [85]. Treg with *MCT1* deletion had reduced lactate uptake and lower proliferation rate in melanoma tumors [85]. Treatment of MCT inhibitors blocked lactate export by cancer cells and these inhibitors synergized with anti-PD-1 therapy to profoundly promote IFNγ production by CD8^+^ T cells, thereby reducing tumor burden [86]. Notably, MCT inhibition did not affect viability and effector function of T cells. *MCT1* deletion in Treg synergized with anti-PD-1 antibodies to promote tumor regression and significantly increased the survival of tumor-bearing mice [85]. Moreover, knockout of *LDHA* ameliorated the efficacy of anti-PD-1 antibodies to markedly increase infiltrated CD8^+^ T cells and NK cells and reduced Treg in a melanoma mouse model [87], suggesting the potential of targeting lactate metabolism to improve the effectiveness of ICIs in hypoxic tumors.

### 3.3. HIF-Mediated Suppression of Mitochondrial Metabolism under Hypoxia

Mitochondrial activities are suppressed to prevent excessive ROS accumulation under hypoxia. HIF-1 upregulated PDK1 in hypoxic cancer cells (Table 1) [44,45]. PDK1 inactivates (pyruvate dehydrogenase) PDH enzyme complex, thereby inhibiting the conversion of pyruvate to acetyl-CoA, the substrate of TCA cycle [24,25] (Figure 3). Knockdown of *PDK1* led to reduction of pyruvate, but increased levels of TCA intermediates under hypoxia, consolidating the role of PDK1 as a negative regulator of TCA cycle [88]. Loss of PDK1 increased mitochondrial O_2_ consumption and induced apoptosis especially under hypoxia [44,45,89], whereas overexpression of PDK1 promoted PDH E1α subunit phosphorylation and rescued survival of HIF-1α knockout cells cultured under hypoxia [44]. Furthermore, HIF-1 elicited a switch of ECT complex I subunit from NADH dehydrogenase (ubiquinone) 1 alpha subcomplex 4 (NDUFA) to NADH dehydrogenase (ubiquinone) 1 alpha subcomplex 4–like 2 (NDUFA4L2), a less active subunit, to decelerate electron transfer and attenuate ROS production in HCC [46,90] (Figure 3) (Table 1). Knockdown of *NDUFA4L2* suppressed HCC tumor growth and lung metastasis [46]. These tumors also had higher oxidative stress, indicating the importance of NDUFA4L2 in lowering ROS in hypoxic HCC. Similarly, HIF-1 modulated a switch from cytochrome C oxidase subunit IV isoform 1 (COX4-1) to COX4-2, a less active subunit in ETC complex IV to prevent excessive ROS accumulation under hypoxia [47] (Figure 3) (Table 1). HIF-1 simultaneously upregulated COX4-2 and mitochondrial protease LON peptidase, which degrades COX4-1 subunit [47]. Both knockdown of *COX4-2* and overexpression of *COX4-1* induced oxidative stress in hypoxic cancer cells [47]. Moreover, HIF-1 induced *miR-210*, which suppresses iron-sulfur cluster assembly enzyme 1/2 (ISCU1/2) in complex I and III to reduce mitochondrial activity and ROS production under hypoxia [48] (Table 1). Clinically, overexpression of PDK1, NDUFA4L2 and *miR-210*, as well as the downregulation of COX4-1 were correlated with poor overall survival of HCC patients [46,50,51,91].

Mitochondrial biogenesis is also suppressed in HCC cells under hypoxic stress. HIF-1 induced hes related family BHLH transcription factor with YRPW motif 1 (HEY1), a transcriptional repressor in the Notch signal pathway, that downregulate PTEN induced kinase 1 (PINK1) essential for mitochondrial biogenesis (Table 1) [49]. *HEY1* overexpression or *PINK1* knockdown reduced mitochondrial mass, mitochondrial cristae structure and ROS level in hypoxic HCC cells [49]. In vivo, *HEY1* overexpression or *PINK1* knockdown consistently promoted HCC growth, demonstrating the protumoral effect of the HIF-1/HEY1/PINK1 axis in hypoxic HCC [49]. Moreover, HIF-1 suppressed MYC proto-oncogene, BHLH transcription factor (c-MYC) signaling, which had been implicated for mitochondrial biogenesis. HIF-1-induces MAX-interacting protein 1 (MXI-1), which competes with MAX for binding with c-MYC to repress c-MYC activity, in hypoxic cancer cells (Table 1) [52]. While MYC-MAX heterodimers transcriptionally activate target genes, MYC-MXI results in transcription repression. Consequently, peroxisome proliferator-activated receptor γ coactivator 1β (PGC-1β), a c-MYC target important for mitochondrial biogenesis, was downregulated to lower mitochondrial activity and ROS level [52]. Overexpression of HEY1, downregulation of PINK1 and PGC-1β expression were commonly found in HCC patients [49,92].

#### HIF-Mediated Suppression of Mitochondrial Metabolism under Hypoxia and TKI Resistance

HIFs suppressed mitochondrial metabolism to limit ROS production under hypoxia. Importantly, Sorafenib induced ROS in HCC cells [29,93]. Antioxidant treatment partially rescued cell death upon Sorafenib treatment, suggesting that Sorafenib induced cell death at least partially by inducing oxidative stress. Sorafenib treatment also elevated serum level of advanced oxidation protein products (AOPP), a marker of oxidative stress, in HCC patients [29]. Notably, Sorafenib-treated HCC patients with high AOPP manifested better drug response and survival benefits [29]. It is reasonable to speculate that HIF-mediated suppression of mitochondrial metabolism contributes to Sorafenib resistance by lowering ROS in drug-treated hypoxic HCC. High mitochondrial activity sensitized multiple HCC cell lines to Sorafenib treatment [61]. PDK1 reduces the rate of TCA cycle to prevent ROS accumulation in hypoxic cancer cells. Dichloroacetate (DCA), a PDK inhibitor, greatly sensitized HCC cells to Sorafenib treatment (Table 2) [61].

### 3.4. HIF-Mediated Induction of Serine Metabolism under Hypoxia

In SSP, glucose-derived 3PG, is converted into serine via a three-step enzymatic reaction catalyzed by phosphoglycerate dehydrogenase (PHGDH), phosphoserine aminotransferase 1 (PSAT1) and phosphoserine phosphatase (PSPH). Serine is the substrate of folate cycle, which generates a key antioxidant, NADPH. Serine hydroxymethyltransferase 2 (SHMT2) first converts serine and tetrahydrofolate (THF) to glycine and methylene tetrahydrofolate (MTHF). Other enzymes in the folate cycle include methylenetetrahydrofolate dehydrogenase (NADP+ dependent) 2 (MTHFD2), MTHFD1L and aldehyde dehydrogenase 1 family member L1/2 (ALDH1L1/2). SSP and its downstream folate cycle are activated under hypoxia in an HIF-dependent manner, producing NADPH to counteract oxidative stress in hypoxic cancer cells [28]. PHGDH and SHMT2 were consistently induced in all six breast cancer cells lines cultured under hypoxia, while PSAT1, PSPH, MTHFD2 and MTHFD1L were induced in most of the cell lines in a HIF-dependent manner (Table 1) [28]. Knockdown or inhibition of PHGDH, the first rate-limiting enzyme in SSP, dramatically reduced the level of NADPH, increased level of mitochondrial ROS and induced more extensive cell death in hypoxia [28,94]. Knockdown of *PHGDH* reduced the level of serine but led to accumulation of glycolytic metabolites, demonstrating that 3PG from glycolysis was diverted to SSP in hypoxic cancer cells [28]. In vivo, PHGDH was overexpressed in hypoxic regions of breast tumors [28]. Tumors derived from *PHGDH* knockdown cells grew much slower with higher level of mitochondrial ROS [28]. SHMT2 was also induced by HIF-1 under hypoxia in neuroblastoma cells [95]. IHC study revealed a positive correlation between SHMT2 and HIF-1α protein expression in human neuroblastoma tissues [95]. Knockdown of *SHMT2* reduced ^13^C-glycine level in hypoxic neuroblastoma cells labelled with ^13^C-serine, consolidating the function of SHMT2 in converting serine to glycine [95]. *SHMT2* knockdown reduced level of NADPH, elevated intracellular ROS and induced apoptosis under hypoxia [95]. Important genes in the SSP and folate cycle, including *PHGDH*, *PSPH*, *MTHFD2*, *MTHFD1L* and *SHMT2*, were upregulated in human HCC tissues and their overexpression was associated with poor prognosis in HCC patients [53,54,55,56].

#### HIF-Mediated Induction of Serine Metabolism under Hypoxia and TKI Resistance

Our group employed genome-wide CRISPR/Cas9 knockout library screening and identified *PHGDH* as the most crucial gene contributing to Sorafenib resistance in HCC cells [65]. RNA-seq revealed that SSP was activated and *PHGDH* was significantly overexpressed in the Sorafenib-resistant HCC cell line [65]. Knockdown of *PHGDH* significantly reduced NADPH/NADP^+^ ratio, induced intracellular ROS and mitochondrial ROS as well as induced apoptosis in Sorafenib-treated HCC cells [65]. Tumors formed by *PHGDH* knockdown HCC cells were remarkably more sensitive to Sorafenib [65]. Excitingly, we found that SSP was not only activated by Sorafenib, but also other TKIs, including Regorafenib and Lenvatinib. Regorafenib or Lenvatinib significantly induced expressions of genes in SSP, including *PHGDH*, *PSAT1* and *PSPH*, in HCC cells [65]. PHGDH inhibitor, NCT-503 synergized with Sorafenib to further promote apoptosis in Sorafenib resistant HCC cells (Table 2) [65]. Notably, administration of antioxidant N-acetyl cysteine (NAC) partially rescued cell death caused by combination treatment [65], indicating that NCT-503 sensitized HCC cells to Sorafenib at least partially through inducing oxidative stress. In vivo, combination treatment of NCT-503 with Sorafenib completely suppressed growth of HCC tumors [65]. Astonishingly, apart from Sorafenib, NCT-503 synergized with other HCC TKIs to drastically induce apoptotic HCC cell death (Table 2) [65]. PHGDH is an attractive therapeutic target to overcome TKI resistance in hypoxic HCC. Since SSP fuels the folate cycle to produce antioxidant to counteract ROS in hypoxic tumors, it is worthwhile to explore the potential of genes in the folate cycle as therapeutic targets to re-sensitize TKI-resistant HCC tumors.

### 3.5. HIF-Mediated Induction of Adenosinergic Metabolism under Hypoxia

Hypoxia is associated with a significant increase of ATP level in extracellular space [96]. Extracellular ATP is converted to adenosine by HIF-induced ectoenzymes, CD39/CD39L1 and CD73 (Table 1) [57], leading to the accumulation of adenosine under hypoxia [97] (Figure 4). While the concentration of extracellular adenosine ranges between 10–100 nM in normal tissues, it surges to 9–13 µM in hypoxic tumors [98]. Both adenosine monophosphate (AMP) and adenosine are immunosuppressive metabolites. We previously demonstrated that HIF-1 induced CD39L1 to produce more extracellular AMP, which inhibited differentiation of MDSC, leading to the accumulation of immunosuppressive MDSC in hypoxic HCC [58]. Upon conversion of AMP to adenosine by CD73 in tumor cells, adenosine mediates its immunosuppressive effect by binding to adenosine receptors, A2A receptor (A2AR) and A2B receptor (A2BR) on immune cells. CD73 and A2AR reduced viability, cytotoxicity and IFNγ expression in tumor-infiltrating NK cells and CD8^+^ T cells [99,100,101,102,103]. A2BR signaling was responsible for the enrichment of MDSC and the shift from M1-like to M2-like macrophage [104,105]. Genetic deletion or inhibition of CD39L1, CD73, A2AR or A2BR consistently suppressed tumor growth in vivo, indicating the protumoral role of adenosinergic metabolism and signaling [58,100,101,104]. CD39L1 and CD73 were overexpressed in HCC patients with poor prognosis [58,59].

#### HIF-Mediated Induction of Adenosinergic Metabolism under Hypoxia and ICI Resistance

The immunosuppressive role of adenosinergic signaling provides rationale for combination treatment of drugs targeting adenosine metabolism to overcome ICI resistance in HCC. We showed that CD39L1 inhibitor, POM-1, synergized with ICIs to increase lymphocyte infiltration, suppress tumor growth and promote survival of HCC-bearing mice (Table 2) [58]. Excitingly, anti-CD39 (IPH5201) and anti-CD73 (IPH5301) monoclonal antibodies have recently been developed [106]. Inhibiting CD39 and CD73 by these antibodies effectively repressed AMP- or adenosine-mediated suppression of CD8^+^ T cell proliferation and greatly promoted antitumor immune response in vivo [106]. Anti-CD39 and anti-CD73 antibodies synergized with each other to activate CD8^+^ T cells and promote antitumor immunity by abrogating adenosinergic signaling [106]. Excitingly, combination treatment of anti-CD39 or anti-CD73 antibodies with anti-PD-L1 antibody in subjects with advanced solid tumors is currently undergoing Phase 1 clinical trial (NCT04261075). Inhibitors or antibodies targeting adenosine receptors, A2AR and A2BR will be useful to study whether inhibition of adenosine sensing by intratumoral immune cells can further sensitize hypoxic HCC to ICIs treatment.

## 4. Targeting Hypoxic HCC to Overcome Drug Resistance

HIF-induced metabolic reprograming is one of the major molecular mechanisms that contributes to TKI and ICI resistance in HCC. Inhibitors suppressing HIFs and HIF-induced metabolic genes are attractive candidates to be targeted in combination with TKIs and ICIs in HCC patients. Furthermore, targeting hypoxic HCC cells in a HIF-independent manner represents a new direction for HCC therapeutic intervention to suppress the molecular events elicited by hypoxia. An elegant drug screening identified digoxin, an antitumor HIF-1 inhibitor that suppressed growth and progression of hypoxic tumors, including HCC [107,108,109]. Another HIF-1 inhibitor, EF24 synergized with Sorafenib to reduce cell viability and promote apoptosis of hypoxia HCC cells in vitro and suppressed HCC growth and lung metastasis in vivo [62]. However, the efficacy of Digoxin and EF24 in human HCC is unknown. Following the discovery of targetable PAS-B domain in HIF-2α, PT2385, an HIF-2 inhibitor, was identified [110]. PT2385 synergized with Sorafenib to inhibit HCC growth in vivo [111]. Nonetheless, as renal cancer cells eventually developed resistance to HIF-2 inhibitors [112], more studies are needed to investigate if resistance to PT2385 will develop in HCC-bearing mice and HCC patients. Two groups reported the tumor suppressive role of HIF-2α in HCC [113,114]. Although the majority of studies confirmed HIF-2α as an oncogene and a prognostic marker associated with poor prognosis in HCC patients [115,116,117], further investigation is necessary to reconcile the opposing results. Interestingly, functional screening has identified HIF-independent genes important for hypoxic HCC cell survival, which could be exploited as therapeutic targets. Our group employed genome-wide CRISPR/Cas9 library screening and identified protein-tyrosine phosphatase mitochondrial 1 (PTPMT1) as a crucial metabolic regulator for survival of HCC cells under hypoxia [118]. PTPMT1 synthesizes cardiolipin, which is the major constituent of the mitochondrial inner membrane (MIM), which anchors the ETC complexes to allow efficient electron transfer. Inhibition of PTPMT1 led to excessive electron leakage at the ETC, rendering cell death in hypoxic HCC cells. PTPMT1 inhibitor significantly suppressed growth and progression of hypoxic HCC [118], making it a promising drug for combination treatment to combat drug resistant HCC. Genes that are functionally important for hypoxic cell survival might not necessarily be induced by HIFs therefore could also be attractive therapeutic targets to overcome drug resistance in hypoxic HCC.

## 5. Conclusions

Hypoxia is an important component of the TME in HCC. Hypoxia elicits metabolic alterations via HIFs which contribute to resistance of existing HCC therapies, leading to dismal therapeutic outcomes. Overcoming drug resistance in hypoxic HCC is a high priority to ameliorate quality of patients’ lives. Targeting genes in metabolic pathways that are rewired under hypoxia opens new hope to overcome drug resistance in HCC. While most studies investigated the roles of metabolic reprogramming under hypoxia in conferring Sorafenib resistance, there is a lack of knowledge whether these metabolic rewiring also contribute to resistance of other FDA-approved TKIs for HCC, including Lenventinib, Cabozantinib, Regorafenib and Ramucirumab. It also remains elusive whether inhibitors that target metabolic genes that are induced by HIFs under hypoxia can synergize with other TKIs, apart from Sorafenib, to increase their therapeutic benefits in treating HCC. Furthermore, more preclinical studies are urgently needed to study whether targeting HIF-induced lactate and adenosine metabolism can ameliorate the efficacy and response rate of ICIs in HCC models. However, the potential side effects of metabolic inhibitors to metabolism and cytotoxicity of anti-tumoral immune cells should be carefully evaluated to determine an appropriate therapeutic window of combination treatment with ICIs to combat hypoxic HCC. More translational efforts are warranted to evaluate the efficacy of combining different inhibitors targeting HIF-induced metabolic genes with TKIs or ICIs to overcome drug resistance in HCC patients.

## Figures and Tables

**Table 1:** Hypoxia-induced alterations of different metabolic pathways, their associated genes and their clinical relevance in human HCC.

Metabolic Pathways	Genes	HIF-Inducible	Expression in Human HCC
**Glucose Metabolism** (Activated)	*GLUT1*, *GLUT3*, *HK2*, *ALDA* and *GAPDH*	Yes [30]	Overexpressed [19,32,36,37,38,39,40]
	*PFKL*, *ALDC*, *TPI*, *PGK1*, *ENO1* and *PKM*	Yes [30]	Undetermined
**Lactate Metabolism** (Activated)	*LDHA* and *MCT4*	Yes [26,41,42]	Overexpressed [20,43]
**Mitochondrial Metabolism** (Suppressed)	*PDK1*, *NDUFA4L2*, *COX4-2*, *miR-210*, *HEY1*	Yes [44,45,46,47,48,49]	Overexpressed [46,50,51]
	*MXI-1*	Yes [52]	Undetermined
**Serine Synthesis Pathway and Folate Cycle** (Activated)	*PHGDH*, *PSPH*, *SHMT2*, *MTHFD2* and *MTHFD1L*	Yes [28]	Overexpressed [53,54,55,56]
	*PSAT*	Yes [28]	Undetermined
**Adenosinergic Metabolism** (Activated)	*CD39*/*CD39L1* and *CD73*	Yes [57]	Overexpressed [58,59]

**Table 2:** Combination treatments of inhibitors targeting genes in metabolic pathways altered by HIFs with TKIs or ICIs to overcome drug resistance in hypoxic HCC.

Inhibitors	Targets	Combination Treatment	Synergistic Effects on Hypoxic HCC
**2-DG**	Glycolysis	Sorafenib (TKI)	Reduced cell viability, induced oxidative stress and apoptosis [63,64]
**3-BP**	HK2 (glycolysis)	Sorafenib (TKI)	Reduced cell viability in vitro and suppressed HCC progression in vivo [40]
**DCA**	PDK1 (mitochondrial activity)	Sorafenib (TKI)	Promoted apoptosis and induced oxidative stress in vitro and suppress HCC progression in vivo [61]
**NCT-503**	PHGDH (SSP)	Sorafenib, Regorafenib and Lenvatinib (TKIs)	Promoted apoptosis and induced oxidative stress in vitro and suppressed HCC progression in vivo [65]
**POM-1**	CD39L1 (adenosinergic metabolism)	Anti-PD-1 and anti-CTLA-4 antibodies (ICIs)	Promoted lymphocyte infiltration and suppressed HCC progression in vivo [58]

## Data Availability

Not applicable.

## Abbreviations

A2A receptor (A2AR); acetyl coenzyme A (acetyl-CoA); adenosine monophosphate (AMP); adenosine triphosphate (ATP); advanced oxidation protein products (AOPP); aldehyde dehydrogenase 1 family member L1 (ALDH1L1); aldolase (ALD); arterial chemoembolization (TACE); arterial embolization transcatheter (TAE); carbonic anhydrase 9 (CA9); clustered regularly interspaced short palindromic repeats (CRISPR); CRISPR associated protein 9 (Cas9); cytochrome C oxidase (COX4); Dichloroacetate (DCA); electron transport chain (ETC); enolase 1 (ENO1); fibroblast growth factor (FGF); flavin adenine dinucleotide (FADH2); fluorodeoxyglucose (FDG); Food and Drug Administration (FDA); glucose transporter type 1 (GLUT1); glucose transporter type 3 (GLUT3); G protein-coupled receptor (GPR); glyceraldehyde-3-phosphate dehydrogenase (GAPDH); hepatocellular carcinoma (HCC); hes related family BHLH transcription factor with YRPW motif 1 (HEY1); hexokinase 2 (HK2); hypoxia-inducible factor (HIF); immune checkpoint inhibitors (ICIs); interferon gamma (IFNγ); iron-sulfur cluster assembly enzyme (ISCU); lactate dehydrogenase A (LDHA); MAX-interacting protein 1 (MXI-1); median partial pressure of O_2_ (pO_2_); methylene tetrahydrofolate (MTHF); methylenetetrahydrofolate dehydrogenase (NADP+ dependent) 1 like (MTHFD1L); methylenetetrahydrofolate dehydrogenase (NADP+ dependent) 2,(MTHFD2); microRNA-210 (*miR-210*); mitochondrial inner membrane (MIM); monocarboxylate transporter (MCT); MYC proto-oncogene, BHLH transcription factor (c-Myc); myeloid-derived suppressor cells (MDSC); N-acetyl cysteine (NAC); nicotinamide adenine dinucleotide phosphate (NADPH); NADH dehydrogenase (ubiquinone) 1 alpha subcomplex 4 (NDUFA); natural killer (NK); NADH dehydrogenase (ubiquinone) 1 alpha subcomplex 4–like 2 (NDUFA4L2); nicotinamide adenine dinucleotide (NADH); oxidative phosphorylation (OXPHOS); peroxisome proliferator-activated receptor γ coactivator 1β (PGC-1β); phosphofructokinase, liver type (PFKL); phosphoglycerate dehydrogenase (PHGDH); phosphoglycerate kinase 1 (PGK1); phosphoserine aminotransferase 1 (PSAT1); phosphoserine phosphatase (PSPH); platelet-derived growth factor (PDGF); protein tyrosine phosphatase mitochondrial 1 (PTPMT1); PTEN induced kinase 1 (PINK1); pyruvate dehydrogenase (PDH); pyruvate dehydrogenase kinase 1 (PDK1); pyruvate kinase M1/2 (PKM); reactive oxygen species (ROS); regulatory T cells (Treg); serine hydroxymethyltransferase 2 (SHMT2); serine synthesis pathway (SSP); solute carrier family 2 member 1 (SLC2A1); solute carrier family 2 member 3 (SLC2A3); tetrahydrofolate (THF); transcriptional enhanced associate domain (TEAD); tricarboxylic acid cycle (TCA); triosephosphate isomerase 1 (TPI1); tumor microenvironment (TME); tyrosine kinase inhibitor (TKI); vascular endothelial growth factor (VEGF); WW domain containing transcription regulator 1 (TAZ); 2-Deoxyglucose (2-DG); 3-bromopyruvate (3-BP); 3-phosphoglyceric acid (3PG).
